# Drug-Induced Liver Injury Due to Doxycycline: A Case Report and Review of Literature

**DOI:** 10.7759/cureus.59687

**Published:** 2024-05-05

**Authors:** Nikola Nikolajevic, Milan Nikolajevic, Ivana Pantic, Bojan Korica, Magdalena Kotseva, Tamara Alempijevic, Dorde Jevtic, Cristian I Madrid, Igor Dumic

**Affiliations:** 1 Internal Medicine, University of Belgrade, Faculty of Medicine, Belgrade, SRB; 2 Gastroenterology and Hepatology, Clinic for Gastroenterology, University Clinical Center of Serbia, Belgrade, SRB; 3 Internal Medicine, Franciscan Health, Olympia Fields, USA; 4 Internal Medicine, NYC Health + Hospitals/Elmhurst, Queens, USA; 5 Internal Medicine, Mayo Clinic Health System, Eau Claire, USA; 6 Hospital Medicine, Mayo Clinic Health System, Eau Claire, USA

**Keywords:** cholestatic liver injury, hepatocellular liver injury, lyme disease and other tick borne pathogens, : doxycycline, drug-induced liver injury (dili)

## Abstract

Antibiotics are among the most common causes of drug-induced liver injury worldwide. Amoxicillin/clavulanic acid and nitrofurantoin are the most common culprits while tetracyclines are a rare cause of liver injury. Among tetracyclines, minocycline has been reported more frequently than doxycycline, which is an extremely rare cause of drug-induced liver injury. We present a healthy 28-year-old male patient from rural United States who was taking doxycycline for Lyme disease. After five days of therapy, he developed nausea, vomiting, fatigue, and significant transaminitis consistent with a hepatocellular pattern of liver injury. After a thorough workup which ruled out other causes such as infection, autoimmune diseases, liver malignancy, and vascular, structural, and metabolic disorders, his liver injury was attributed to doxycycline. We reached the diagnosis also by demonstrating a consistent temporal association between doxycycline intake and liver injury and the patient recovered completely with the cessation of doxycycline. Recognition of doxycycline as a cause of drug-induced liver injury should be considered in patients utilizing this antibiotic. Doxycycline, unlike minocycline, has a short latency period. Early recognition and discontinuation of doxycycline in our patient resulted in the complete resolution of symptoms and transaminitis preventing further morbidity and mortality.

## Introduction

Drug-induced liver injury (DILI) is a relatively rare condition that develops due to the use of prescription medications, dietary and herbal supplements, and illicit drugs [1-3]. Traditionally DILI has been classified as intrinsic (direct) and idiosyncratic. Intrinsic DILI is dose-dependent, predictable, and often has a short latency period (measured in hours or days). Idiosyncratic DILI is dose-independent and has a variable latency period (days or weeks). Duration of treatment is not considered a risk factor, although the majority of DILI occurs within the first six months from the drug initiation [4].

In the past, the most common drugs that caused DILI in the United States (US) were antiepileptics and antitubercular drugs [4]. With the decreasing incidence of tuberculosis in the US, and the development of biological agents (like infliximab) and immune-checkpoint inhibitors, the tide has shifted, and these medications are now more common causes of DILI [4]. In China and Korea, the most common DILI culprits are herbal and dietary supplements, and ayurvedic medicine products are the main culprit in India [4-6]. Until recently the real incidence of DILI was unclear due to insufficient data obtained in retrospective studies, difficulties in performing causality assessments in medical records, underreporting, and misdiagnosed cases. Recent prospective studies and DILI registries demonstrated that the incidence varies worldwide: the lowest DILI incidence is reported in the US, Iceland, and France (between 14-19 cases per 100.000 inhabitants), and the highest incidence is reported in China with 23.8 cases/100.000 [4,7,8].

Doxycycline is a broad-spectrum antibiotic that belongs to the tetracycline family and is usually prescribed for the treatment of acute bronchitis, community-acquired pneumonia, *Helicobacter pylori* infection, Lyme disease, and some sexually transmitted diseases [9]. Besides its bacteriostatic activity against a variety of bacteria, it also has anti-inflammatory and immunomodulatory effects, particularly for patients with chronic obstructive lung disease [10]. Doxycycline is usually well tolerated. The most common side effects include nausea, epigastric pain, and antibiotic-associated diarrhea. Several studies affirm that prolonged administration of subantimicrobial doses of doxycycline does not impact the resistance of the host microbiota [11]. Dose adjustment is usually not needed in patients with renal and liver impairment. Studies have shown that doxycycline has lower hepatotoxicity than tetracycline and that doxycycline-induced DILI is an exceedingly rare side effect [12].

## Case presentation

A 28-year-old male farmer from rural Wisconsin presented to the emergency department complaining of one day of nausea, vomiting, and fatigue. He denied abdominal pain, hematemesis, or fever. Six days prior to presentation, he was diagnosed with Lyme disease based on the findings of erythema migrans and positive epidemiological data, as he was bitten by a tick. At the time of diagnosis, routine complete blood cell count (CBC) and comprehensive metabolic panel (CMP) were checked and all laboratory parameters were within normal range, including liver function tests (LFTs). The patient was prescribed doxycycline 100 mg tablets two times daily for two weeks for early localized Lyme disease. The patient discontinued doxycycline on his own on day 5 due to nausea. He denied any alcohol consumption, was not a smoker, and denied the use of any illicit drugs. He was in a monogamous heterosexual relationship and denied risks for sexually transmitted infections. The patient did not take any other medications, herbal supplements, or over-the-counter remedies.

Initial assessment revealed a well-developed male patient in no apparent distress. His body mass index (BMI) was 20.5 kg/m^2^. His vital signs were normal. His mucous membranes were dry. Examination of the heart, lungs, and abdomen was normal. Erythema migrans resolved and no other skin changes were noted. Examination of the joints did not show any evidence of redness, swelling, or pain during passive and active motions. Blood work demonstrated normal CBC, with significant elevation of liver enzymes. Specifically, aspartate aminotransferase (AST) was 814 IU/L (reference, <20 IU/L) and alanine transaminase (ALT) was 758 IU/L (reference, <20 IU/L). International normalized ratio was 1.1 (normal) and total bilirubin was 0.6 mg/dL (normal). All other laboratory parameters were within normal range.

Further diagnostic workup was carried out including serology and polymerase chain reaction (PCR) for human immunodeficiency virus (HIV), cytomegalovirus (CMV), and Epstein-Barr virus (EBV), which was negative. Hepatitis A virus (HAV), IgM, and IgG serology were negative, hepatitis B virus (HBV) antigen was negative, HBV surface antibody was positive (vaccinated), and hepatitis C virus (HCV) IgM and IgG antibodies were negative. Abdominal ultrasonography, including Doppler assessment of portal blood vessels, was negative for liver steatosis, liver masses, biliary obstruction, or vascular liver disease. Autoimmune workup including complement levels, anti-smooth muscle antibodies, antimitochondrial antibodies, and antinuclear antibody testing returned normal. Testing for celiac disease was negative as well. By excluding other infectious, malignant, vascular, and autoimmune liver diseases, diagnosis of hepatocellular pattern of idiosyncratic DILI due to doxycycline was established.

For the next 48 hours, the patient remained nauseous, and vomiting was controlled by the use of IV promethazine. He remained hemodynamically stable and afebrile. The trend of liver enzymes is illustrated inFigure1*.*

**Figure 1:**
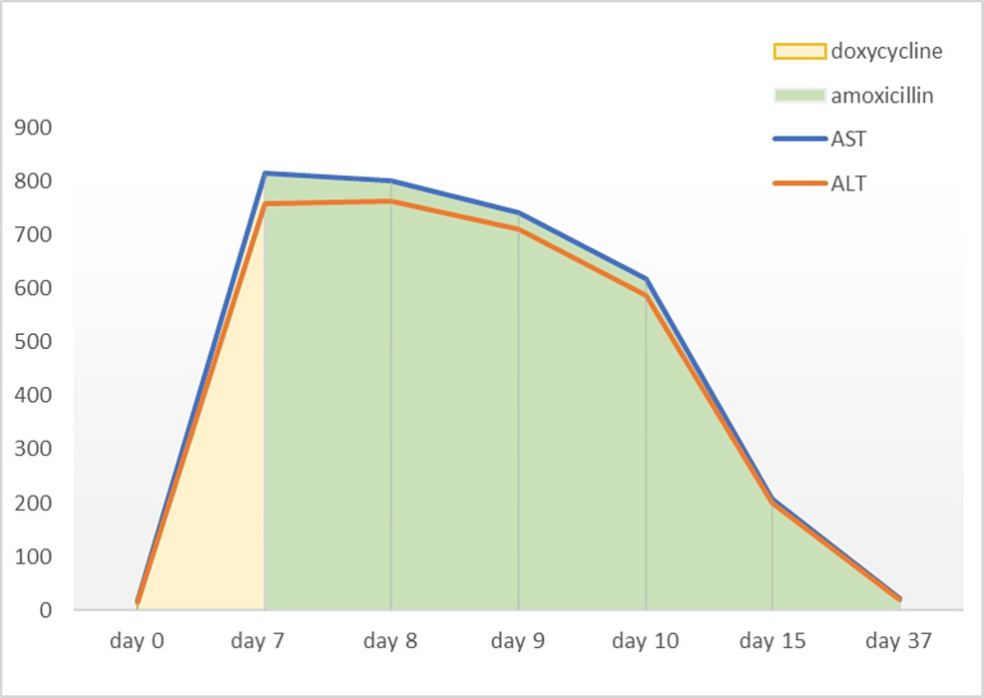
Liver enzyme abnormalities during doxycycline therapy and their normalization with cessation of therapy when patient was switched to amoxicillin AST: aspartate aminotransferase; ALT: alanine aminotransferase. Unit of measurement is U/L.

After 72 hours, the patient was discharged home on oral amoxicillin to complete a total of 14 days of treatment, which he tolerated well. Within one month from the time of diagnosis, liver enzymes were all within the normal range.

## Discussion

Antibiotics are the most common class of medication causing DILI, and some of them are more likely than others to be the culprit. A study on DILI done in Iceland found that amoxicillin-clavulanate was the most common cause, followed by nitrofurantoin, while doxycycline was the causative agent in only two out of 32 677 cases [13]. Our patient presented with a hepatocellular pattern of liver injury that has improved with doxycycline withdrawal. We reached the diagnosis by demonstrating a consistent temporal association to doxycycline intake and liver injury, and by ruling out other diseases that can present similarly. Additionally, the Roussel Uclaf Causality Assessment Method (RUCAM) score for this patient was 8, while on the Naranjo Adverse Drug Reaction Probability Scale, he scored 6, defining this case as “probable” DILI.

We performed a search of the PubMed/MEDLINE (Medical Literature Analysis and Retrieval System Online) database using the following keyword combination: doxycycline, minocycline, tigecycline, tetracycline and liver injury and/or DILI. The search yielded eight additional cases, which are presented in more detail in Table 1*.* Out of these eight, three DILI cases were due to doxycycline, one due to tetracycline, one due to tigecycline, and the remaining cases were due to minocycline. We excluded from the search cases of autoimmune hepatitis due to minocycline and cases where DILI was part of a drug reaction with eosinophilia and systemic symptoms (DRESS) syndrome as these are separate entities from pure idiosyncratic DILI.

**Table 1:** Previously reported cases of idiosyncratic DILI due to doxycycline, tigecycline, minocycline, and tetracyclines. Cases of DRESS syndrome DILI and autoimmune hepatitis were not included as they are separate entities. BID: two times a day; LFT: liver function test; M: male; F: female; PO: per os (orally); IV: intravenous; DILI: drug-induced liver injury; DRESS: Drug Reaction with Eosinophilia and Systemic Symptoms

Case	Drug	Reference	Age	Sex	Dose/formulation/regimen	Latency/ time to injury	Outcome	DILI pattern	Biopsy	Steroids	Special comment
1	Doxycycline	Varma et al. (2021) [14]	43	F	100 mg PO BID	10 days	LFT normalized	cholestatic	Mild mixed inflammatory infiltrate composed of lymphocytes, occasional eosinophils and neutrophils, and rare plasma cells	No	
2	Doxycycline	Lienart et al. (1992) [15]	42	F	200 mg PO Daily	5 days	LFT normalized	cholestatic	No	No	
3	Doxycycline	Westermann et al. (1999) [16]	38	M	1000 mg PO Daily	12 years	LFT normalized	hepatocellular	Fatty degeneration, hepatocellular necrosis, and cholestasis	No	
4	Tetracycline	Glenn et al. (2011) [17]	49	F	500 mg PO BID	7 months	LFT normalized	cholestatic	Mild lymphocytic inflammation within the portal tract areas	No	
5	Tigecycline	Shi et al. (2022) [18]	68	M	100mg IV BID	5 days	LFT normalized	cholestatic	Cholestasis, micro cavitation, and punctate necrosis of liver cells	Yes (methylprednisolone)	Post-transplant DILI
6	Minocycline	Parker et al. (2018) [19]	32	M	100 mg PO Daily	7 months	LFT normalized	hepatocellular	No	Yes (prednisone)	
7	Minocycline	Shankar et al. (2018) [20]	15	F	50 mg PO BID	4 weeks	LFT normalized	hepatocellular	Acute portal and lobular hepatitis with lymphocytic and abundant eosinophilic infiltrate	No	
8	Minocycline	Rikken et al. (2004) [21]	28	M	100 mg PO Daily	2 years	LFT normalized	hepatocellular	No	Yes (prednisone)	

Patterns of injury and latency

DILI associated with amoxicillin-clavulanate most commonly presents as a cholestatic pattern of liver injury [14,15]. Patients with nitrofurantoin-induced DILI usually develop a hepatocellular pattern of liver injury [16,17]. When cephalosporins are identified as the cause of DILI, the pattern of injury is usually cholestatic or mixed [18]. Doxycycline, minocycline, and tetracycline might cause both hepatocellular and cholestatic patterns of liver injury [19]. Tigecycline can cause all patterns of DILI, although cholestatic is the most common [20-22]. Fluoroquinolones (FQ) cause all three patterns of injury [23]. From macrolides, erythromycin has shown cholestatic and mixed types of DILI, while azithromycin causes predominantly hepatocellular injury [24,25]. Sulfonamides can present with all three types of injury [26].

Latency, defined as the time from medication intake until the development of liver injury, is usually shorter in nitrofurantoin and FQ DILI, but there is no universal rule and exceptions do exist [27]. Doxycycline-induced liver injury usually has a short latency period occurring within one to two weeks of starting doxycycline [28]. One case reported a latency of only five days [29], similar to the case we are reporting. Interestingly, a case report from Germany documented various adverse reactions including DILI in a patient with depersonalization disorder who overmedicated himself chronically with 1 gram of doxycycline daily for 12 years [30]. In this extreme case, all adverse reactions resolved after doxycycline discontinuation. The latency period of cephalosporins-induced DILI is typically longer, about one to three weeks so patients can remain asymptomatic during the treatment course and develop symptoms only days after the treatment has already ended [18]. One case reported a 17-year-old girl who started taking doxycycline for acne and five days later presented with jaundice, vomiting, nausea, headaches, myalgia, and lightheadedness. Subsequently, she was diagnosed with Wilson’s disease and DILI. She underwent successful liver transplantation on the fourth day of hospitalization and recovered fully [31,32]. Given the close temporal relationship between the initiation of doxycycline and the development of liver failure, it was felt that DILI was a “second hit” that precipitated liver failure in the background of undiagnosed Wilson’s disease. From Table 1 we can see that doxycycline is usually associated with very short latency (DILI develops mostly within a week) while minocycline has a longer latency period.

Risk factors

The exact risk factors associated with the development of DILI are not fully clarified yet. For example, age as a risk factor is inconsistent across DILI registries. While changes associated with immunosenescence might play a role, there is a chance that DILI is more common in the elderly due to polypharmacy, which is very prevalent in this age group. The elderly might be at higher risk from isoniazid toxicity, while younger people may be more vulnerable to valproate and aspirin-related toxicities [33]. No difference has been detected in DILI incidence with respect to gender. However, it has been noted that women are more susceptible to developing DILI with autoimmune features and tend to progress to acute liver failure more often once liver injury develops [34]. African Americans may have a higher risk of DILI from trimethoprim/sulfamethoxazole and methyldopa which points toward a genetic predisposition for DILI development. Mutations in genes related to bile salt export can increase the risk of cholestatic DILI [33,34].

Alcohol may not directly cause DILI, but it worsens its prognosis, particularly when the causes of DILI are acetaminophen and methotrexate [33]. There is limited data on DILI during pregnancy, and it is mainly caused by medications used for gestational hypertension such as methyldopa and hydralazine [34]. Chronic liver diseases and viral infections like HCV and HIV can lead to more severe forms of DILI [35-37]. Gut microbiota plays a key role in the body's homeostasis and immunity, and emerging research suggests that derangement in microbiome and bacterial metabolism can increase the risk for DILI, although firm evidence is still missing [38-40].

Clinical presentation

DILI can be asymptomatic and evident only by mild elevation of liver enzymes, can present with features of hepatitis such as fever, abdominal pain, nausea, and vomiting, or can be severe leading to acute liver failure (ALF) and death. Even though exceedingly rare, some patients can develop chronic DILI [41,42]. The range of the symptoms is broad, but the most common appears as a viral-like hepatitis syndrome with nausea, fatigue, and right upper quadrant (RUQ) abdominal pain linked with an elevation in LFTs. When DILI is part of a DRESS syndrome, patients will often have a fever, rash, eosinophilia, lymphadenopathy, and other visceral organ involvement [43]. DILI due to doxycycline can occur with immunoallergic features that is similar to DRESS syndrome [44]. 

Diagnosis

Diagnosing DILI is often challenging. There is no pathognomonic clinical sign or laboratory value that can be used to rule in or rule out DILI. Instead, diagnosis mostly relies on a high index of suspicion. The new revised electronic causality assessment method (RECAM), an improved version of the RUCAM, has recently been proposed and endorsed to help with improved DILI diagnosis [45]. The utilization of RECAM through computerization is crucial due to the limitations faced by RUCAM when used by different individuals. RECAM simplifies the process by automating the assessment, requiring users to input dates and laboratory results. This computerized approach enhances reliability and impartiality [45,46].

DILI is usually a diagnosis of exclusion, and the clinician must exclude all other conditions that may present similarly. Once other potential causes of liver injury are excluded (viral infection, autoimmune diseases, liver malignancy, vascular and metabolic disorders), and consistent temporal association with culprit drug administration taken into account, diagnosis of DILI can be established.

The utility of liver biopsy in cases of DILI holds significant value, especially in situations where diagnosis and causality assessment are complex. While the role of liver histology in managing DILI remains uncertain, it can be highly beneficial particularly, when performed within the first 60 days after the onset of liver injury [45]. Histological analysis increased the likelihood of DILI diagnosis in 48% of cases and confidently excluded DILI in 20% of cases, thereby clarifying diagnoses [45]. Liver biopsy plays a crucial role in cases where differentiation from other conditions like autoimmune hepatitis, non-alcoholic steatohepatitis, and cholestasis [47]. Given its invasiveness, cost, risks, and the rapid normalization of liver enzymes after doxycycline discontinuation, we did not perform a liver biopsy on our patient

Sometimes, it is very difficult to determine the exact cause of DILI. It is especially difficult when the patient is taking multiple antibiotics, for example in resistant *Helicobacter pylori* treatment, which usually involves quadruple therapy. In one case, it was assumed that tetracycline contributed to liver injury but there is no pathognomonic test to prove that [48]. 

Differential diagnosis

DILI has a very broad list of differential diagnoses. Virtually all liver diseases at some point manifest themselves with abnormal LFTs. Some of the most important diseases to exclude are given below.

DRESS Syndrome

The liver is the most common visceral organ involved in patients with DRESS syndrome, and in addition to eosinophilia, LFTs are the most commonly abnormal laboratory findings. Unlike patients with DILI without DRESS syndrome, these patients have fever, lymphadenopathy, and skin rash. Interestingly, DRESS-associated DILI has a lesser degree of LFT derangement and a better prognosis.

Autoimmune Hepatitis (AIH)

AIH can be considered a differential diagnosis for DILI in cases in which liver injury exhibits an autoimmune phenotype. In such cases, even though the relationship between DILI and AIH is complex, it is of great importance to differentiate between the two seemingly similar clinical entities: (i) idiopathic “true” AIH and (ii) drug-induced autoimmune-like hepatitis (DI-ALH), as an idiosyncratic form of DILI. Both entities have immune-mediated pathophysiological mechanisms in common, which are not entirely enlightened. In cases of DI-ALH it is suspected that in some cases, the culprit drugs form neoantigens which stimulate an immune response, which results in liver injury [49]. Several drugs have been reported to be associated with DI-ALH, including minocycline, nitrofurantoin, biological agents (infliximab), statins, and even vaccines [50,51].

The diagnosis of AIH includes a combination of clinical, laboratory, and histological criteria, which are almost always present in patients with DI-ALH [52]. However, since DI-ALH is not expected to develop a chronic form, evidence of advanced chronic liver disease in biopsy specimens could stand in favor of idiopathic AIH [50]. Until now, no differentiating biomarker between the two entities has been found, even though several biomolecules are showing promising results in the early research stages [53].

In the majority of cases, the diagnosis of DI-ALH is established retrospectively. DI-ALH usually responds well to culprit drug withdrawal. Moreover, it has been noticed that DI-ALH patients respond to immunosuppression more promptly and that they usually do not relapse after the immunosuppression withdrawal, which is almost never the case in idiopathic AIH [50].

Viral Hepatitis

Infection with HAV, HBV, HCV, and hepatitis E virus (HEV) must be excluded in each patient who presents with alteration in liver chemistries. Additionally, in specific populations acute HIV should be excluded, as well as acute CMV and EBV infection. Severe acute respiratory syndrome coronavirus 2 (SARS-CoV2) can also cause hepatitis due to its hepatotropism, but patients in addition to LFT abnormalities have other signs and symptoms of the disease. In oncologic and immunocompromised patients checking for herpes simplex virus (HSV) and human herpesvirus 6 (HHV-6) infection should be done as well.

Others

Other differential diagnoses may include: (i) Steatotic liver disease: Patients with elevated BMI, and those with features of metabolic syndrome might have steatohepatitis that can mimic DILI; (ii) Alcohol-related liver disease; (iii) Vascular liver diseases: Portal vein thrombosis or hepatic vein thrombosis can mimic DILI and liver ultrasound with Doppler can be used to diagnose these conditions; (iv) Hepatocellular carcinoma and metastasis to the liver are easily seen by any imaging methodology so this etiology is relatively easy to rule out; (v) Ischemic hepatitis; (vi) Congestive hepatopathy.

Treatment

Prompt recognition of culprit medication and its discontinuation is the main step in managing DILI. In some cases, where DILI is asymptomatic and LFT elevation is mild, but the suspected medication is life-saving (for example, bosentan used for the treatment of pulmonary arterial hypertension) benefit/risk analysis must be individualized for each patient [54].

N-acetyl cysteine (NAC) has been attracting a lot of attention because of its antioxidant capacity and potential to improve intracellular glutathione (GSH) levels. Besides boosting intracellular GSH, the latest findings show that NAC can change other mechanisms of oxidative stress such as decreasing endoplasmic reticulum stress and enhancing mitochondrial function [55]. Pickering et al. showed that a high dose of NAC daily combined with paracetamol could partially counteract paracetamol-induced hepatotoxicity by preserving GSH levels [56]. Interestingly, Morison et al. showed that infusion of NAC with calmangafodipir, a superoxide dismutase mimetic, could decrease biomarkers of paracetamol toxicity such as ALT, keratin-18, and circulating caspase-cleaved cytokeratin 18 [57]. Other than paracetamol overdose, additional studies have reported cases such as injury caused by excessive alcohol intake. Singh et al. exhibited that there was no liver function improvement in cases with severe alcohol hepatitis when adding NAC in patients who take granulocyte colony-stimulating factor (GCSF) [58]. The latter had already shown effectiveness in enhancing liver function and prolonging survival times in these patients. On another hand, a study done by Nabi et al. showed that NAC given in specific doses and infusion timetable reduced mortality and hospital stay in patients with non-paracetamol-induced liver failure [59].

In scenarios such as paracetamol overdose, NAC is a standard treatment, but its effectiveness reduces when administered more than 24 hours after ingestion. In cases of idiosyncratic DILI, limited data support NAC use, but a recent trial showed that NAC is protective in cases of DILI due to antituberculosis drugs and leads to a significant reduction in LFTs at four weeks. Researchers estimating the effect of NAC in preventing liver injury in patients who were treated with ATT drugs observed liver function parameters, oxidative stress biomarkers, and quality of life using the 36-item Short Form Health Survey questionnaire (SF-36). After four weeks of NAC treatment, ALT, ALP, and bilirubin levels significantly reduced and AST lowered by close to 20%. NAC also reduced oxidative stress biomarkers such as malondialdehyde (MDA), nitric oxide (NO), and GSH which were sustained after discontinuation of NAC. Their studies show that simultaneously administering NAC with ATT drugs can reduce ATT-liver injury [54,57,60].

Steroid-Sparing Options for Immune Checkpoint Inhibitor (ICI)-Induced Hepatitis

Corticosteroids are standard therapy for ICI-induced liver injury, with treatment decisions based on liver enzyme levels and DILI severity. ICIs can be continued with close monitoring or they should be temporarily held. Severe cases require permanent discontinuation of checkpoint inhibitors and immediate corticosteroid treatment. However, it should be noted that even corticosteroids can cause DILI [61].

In the treatment of ICI-induced hepatitis, after stopping the treatment with ICI, corticosteroids play a central role, since they are recommended as an initial therapy option. Corticosteroid therapy is not recommended for less severe, such as grade 2 reactions and milder cases, since they can resolve by themselves [62]. According to the findings from a recent systematic review, it was estimated that close to 50% of the 26 patients suffering from severe grade 3-4 ICI-induced hepatotoxicity experienced recovery without the need for corticosteroid intervention [63]. It appears that in most cases of ICI-mediated hepatitis, resolution occurs independently of the corticosteroid dose administration and the efficacy of high-dose corticosteroids in treating other forms of DILIs is still a subject of debate [64].

Prognosis

Most patients with DILI have uneventful courses and full recovery once the offending agent is stopped. Studies have shown that patients with hepatocellular pattern of injury have the highest mortality rates and among them, patients with jaundice are more likely to develop severe disease including ALF and need for transplant. About 10% of patients with DILI develop acute liver failure which is associated with poor survival with mortality rates of 60-80%. AST and bilirubin were found to independently predict death or need for transplantation. Currently, there are no validated prediction scores to determine which patients will progress to ALF [65-67].

In comparison, patients with a cholestatic pattern of injury have a more favorable prognosis but are more likely to develop persistent liver function test abnormalities and progress to a chronic DILI. The mixed pattern carries the most favorable prognosis of all three but is also prone to developing chronic DILI [65]. In a Swedish study, patients with hepatocellular injury had the highest mortality of 12.7%, compared with 7.8 % mortality in the cholestatic injury and 2.4 % in the mixed group [65,66].

Chronic DILI, defined as the presence of LFT abnormalities 6-12 weeks after drug discontinuation, has been reported to occur in 3.4-39% of patients [65-67]. Long-term sequelae of DILI include secondary sclerosing cholangitis (diffuse biliary tract dilatation and thickening seen with cholestatic liver injury), sinusoidal obstruction syndrome (drug-induced vascular injury ), vanishing bile duct syndrome (presents as a bile duct loss on liver biopsy, associated with unfavorable prognosis due to progressive cholestasis leading to liver failure), and as common end stage of all the development of cirrhosis and its complications [66]. Patients with FQ-induced mixed type of liver injury have a better prognosis. Hepatocellular and mixed liver injuries are associated with more severe outcomes while cholestatic type can lead to chronic liver injury.

## Conclusions

Tetracyclines are a rare culprit of DILI, and among them, minocycline is a more common cause than doxycycline. Doxycycline DILI has a very short latency period, measured in days, unlike minocycline which latency is usually longer, and DILI develops after a few weeks of therapy. Patients with deranged LFTs have broad differential diagnoses and DILI is a diagnosis of exclusion. Prompt recognition and discontinuation of the offending medication is the critical step in the management of these patients and contributes to improved morbidity and mortality. Clinicians prescribing doxycycline should be aware of this rare side effect of commonly used medication.
